# Effect of a collaborative multidimensional approach versus usual care on the resolution of adult depression in primary care in Chile: study protocol for a single blinded cluster randomized controlled trial

**DOI:** 10.12688/f1000research.75764.1

**Published:** 2022-02-17

**Authors:** VERONICA VITRIOL, ALFREDO CANCINO, ANDRES SCIOLLA, SERGIO GUIÑEZ, JORGE CALVO, MARCELA ORMAZABAL, JOHANNA KREITHER, SOLEDAD BALLESTEROS, MARIA DE LA LUZ AYLWIN

**Affiliations:** 1 Escuela de Medicina, Universidad de Talca, Talca, Maule, 3460000, Chile; 2 Department of Psychiatry and Behavioral Sciences School of Medicine, UC Davis Medical Center, Sacramento, Californa, 95616, USA; 3 Departamento Atención Primaria, Servicio de Salud del Maule,, Talca, Maule, 3460000, Chile; 4 Centro de Investigación en Ciencias Cognitivas, Universidad de Talca, Talca, Maule, 360000, Chile; 5 Centro de Psicología Aplicada, Universidad Talca, Talca, Maule, 3600000, Chile; 6 Centro de Investigaciones Médicas, Universidad de Talca, Talca, Maule, 3600000, Chile

**Keywords:** Depression treatment, primary care, collaborative care, trauma-informed care, randomized controlled trial

## Abstract

**Background**: Major depression (MD) is a prevalent and disabling condition in Chile. Most MD cases are treated at the primary care level. In Chilean primary care, the authors have gathered evidence of a prevalent complex depression subtype associated with a worse prognosis and characterized by interpersonal difficulties, suicidality and trauma history. This MD presentation suggests the need for a multidimensional, trauma-informed and interprofessional approach.

The present study protocol describes the context, hypotheses and methods for a cluster randomized control trial (RCT) comparing a collaborative multidimensional approach and usual care in treatment outcomes of MD in primary care in Chile.

**Methods**: This is a two-arm, single-blinded, cluster RCT to compare the efficacy of a collaborative multidimensionalapproach for depression (CMAD) versus usual care to treat MD in primary care clinics in Chile. In total, 394 depressed adults from 18 to 65 years of age in twelve clinics located in Chile’s Maule Region will be consented to participate in the study. Patients and care teams from each clinic will be randomized to the intervention or to the control arm.

Interprofessional teams in the intervention arm will attend 27 hours of didactic and active learning sessions focused on clinical competences to effectively engage, treat and follow up patients with complex presentation of MD. Team in the control arm will receive 27 didactic sessions on current clinical guidelines for MD, profesionals in both arms will receive 27 hours of continued education.

**Discussion**: To improve treatment outcomes of MD in Chile, primary care teams should develop clinical competencies relevant to complex, difficult-to-treat types of MD within collaborative and trauma-informed approaches.

## Abbreviations

CMAD: Collaborative multidimensional approach for depression

GAD-7: General Anxiety Disorder Scale-7

GSES: General self-efficacy scale

ERS: Emotion regulation scale

MD: Major depression

MINI: Mini-International Neuro-psychiatric Interview

PHQ9: Patient Health Questionnaire

OQ-45: Outcome Questionnaire 45

RCT: Randomized controlled trial

## Background

Depression is the leading cause of disability worldwide, contributing significantly to the overall global burden of morbidity and mortality. ^1^ It is estimated that it affects almost 280 million people worldwide. ^1^ In Chile, depression constitutes a significant public health problem. ^2^ Findings before the SARS-CoV-2 pandemic showed that 18.2% of the adult Chilean population reported depressive symptoms, and 6.2% met the criteria for major depression (MD). ^3^^,^^4^ This prevalence is expected to increase with the pandemic. ^5^ Furthermore, it affects twice as many women as men, is the second leading cause of disability-adjusted life years and the first among women between 20 and 44 years. ^2^

Since 2001 there has been a Chilean national mental health program to treat MD. From 2006, this program has been known as Explicit Health Guarantees (Garantías Explícitas en Salud, GES, in Spanish), and is regulated by ministerial clinical guideline (Guía Clínica, 2013). ^6^ According to existing data, primary care treat 90% of MD cases. ^7^ Only those with current suicide attempts, suspected bipolarity or psychosis are referred to the specialty level. ^2^^,^^7^

Despite the implementation of the governmental guidelines, evidence shows limited competence in primary care teams to address MD, ^7^ with remission rates close to 50% at one year of follow-up. ^8^^,^^9^ There is also evidence of a high prevalence of comorbid anxiety disorders and reports of adverse life experiences in over 80% of patients with MD. ^10^^,^^11^ Using latent class analysis, our group found evidence of a complex depressive subtype. ^12^ This subtype, whose frequency is about half of the sample studied, is characterized by a history of suicide attempts, history of adverse life experiences, interpersonal difficulties, impairment in social functioning, and poor treatment response. ^12^ These characteristics, which have been reported in refractory depression, ^13^ can be understood in light of the neurobiological sequelae of exposure to psychological trauma. ^14^ Currently, there are a lack of recommendations in depression treatment guidelines regarding the needs of patients with this complex clinical picture.

According to a consensus of international experts, the clinical characteristics of patients with refractory depression are better conceptualized as difficult-to-treat depression. ^13^ This conceptualization implies an understanding of depression as a chronic disease, which requires a collaborative, multidimensional, biopsychosocial clinical approach and an orientation to the management of symptoms and functional recovery. ^13^ Working with similar populations, others have proposed the adoption of trauma-informed care principles and approaches, considering the cumulative interactions between trauma exposure, socioeconomic disadvantage, depression and suicidality ^14^^–^^16^

Both difficult-to-treat depression and trauma-informed care imply a collaborative organization health model similar to that proposed by Wagner *et al* in the management of chronic diseases, ^17^ including anxiety and depressive disorders as proposed by Archer *et al.* ^18^ It involves the presence of a case manager, a structured patient-centered approach, scheduled follow-up visits, the use of validated measures to monitor treatment response, and interprofessional communication between the different levels of care. ^18^ Considering all of the above, we hypothesize that a continuing education program for primary care teams that focuses on a biopsychosocial, trauma-informed care approach implemented in a collaborative model, will improve the outcomes of depression in comparison to usual care.

Specifically, we hypothesize that the collaborative multidimensional approach for depression (CMAD) in primary care will demonstrate greater efficacy relative to the standard treatment based on the clinical guidelines, considering:

1. Adult depressed patients in the CMAD arm will significantly improve depressive symptoms compared to the control group at 3 and 6 months after admission.
2. Adult depressed patients in the CMAD arm will significantly improve anxiety symptoms compared to the control group at 3 and 6 months after admission.
3. Adult depressed patients in the CMAD arm will significantly improve interpersonal dysfunction, social impairment, and emotional dysregulation compared to the control group at 3 and 6 months after admission
4. Adult depressed patients in the CMAD arm will achieve a significantly greater adherence than the control group at 3 and 6 months after admission.

We designed a cluster RCT protocol with two arms, single-blind to the outcome evaluator to compare the efficacy of a CMAD to improve the treatment outcomes of adult depression in primary care in Maule Region in Chile.

The general aim of this study is to compare the efficacy of a CMAD approach for MD with usual care according the standard treatment on the outcomes in primary care clinics in the Maule Region through a cluster RCT. The specific aims of this study are:

1. To design a CMAD treatment approach that imparts primary care teams with the competencies to recognize the clinical, functional and psychosocial dimensions associated with difficult-to-treat depression. This design includes trauma-informed principles and patient-centered care communication skills.
2. To implement the CMAD approach in primary care clinics in the Maule region, including a case manager, a structured patient-centered inter-professional management, a scheduled follow-up using validated instruments and continuous consultation with the specialty level.
3. To compare the depressive symptoms in adults treated for MD in primary care Maule clinics using the CMAD approach versus the standard treatment at baseline, three and six months.
4. To compare the anxiety symptoms in adult depressed patients treated for MD in primary care Maule clinics using the CMAD approach versus the standard treatment at baseline, three and six months.
5. To compare the symptoms of functional variables of emotional regulation, interpersonal and social functions in adult depressive patients treated for MD in primary care Maule clinics, using the CMAD approach versus the standard treatment at baseline, three and six months.
6. To compare the therapeutic adherence in adult depressive patients treated for MD in primary care Maule clinics using the CMAD approach and the standard treatment.

This study protocol is a cluster RCT, two parallel arms, single-blind to the outcome evaluator, which will compare the efficacy of a CMAD to improve the treatment outcomes of adult depression in primary care in the Maule Region in Chile.

## Methods

### Study setting

To meet the objectives of this protocol, primary care clinics belonging to the Maule Region in the municipalities of Talca, Curicó, Constitución, Sagrada Familia, Romeral and Pelarco will be invited to participate. These are centers that represent urban and rural areas of a disadvantaged region, including several rural areas where the socioeconomic and educational level is very low. ^19^ To obtain the sample, 12 centers were identified, which were matched according to similar average socioeconomic and educational characteristics. Moreover, selection considered that there will be no cross contamination between the patients in the control clinics and the intervention clinics. ^34^

### Eligibility criteria

Adults between 18 and 65 years who enter treatment for depression in primary care clinics of the Maule Region will be invited for an evaluation after they sign a written informed consent statement. ^34^ Those with a confirmed diagnosis of MD, according to the Mini-international Neuro-psychiatric interview, will be included in the study. ^20^ On the other hand, those unable or unwilling to sign the informed consent, those with sensory disabilities, no access to a telephone, or who have been referred to a specialist level will be excluded from the study.

### Interventions

Eligible primary care clinics will be randomly assigned to the intervention and control arms of the study using a computer algorithm. Primary care teams of the intervention arm will receive training and implementation of the CMAD approach. Primary care teams of the control group will receive training in-line with the current clinical guideline for depression according.

### Intervention arm

The intervention arm will include 27 hours of training in the CMAD approach for primary care teams of at least one physician, a psychologist and a social worker. This approach integrates knowledge, attitudes, and skills to manage clinical manifestations associated with adversity or trauma throughout the lifespan, which are common in patients with complex difficult-to-treat depression. These competencies will be integrated with, not substituted for, the diagnostic and treatment recommendations included in the current clinical guidelines for depression. The training will be conducted by a team of two professors from the Universidad de Talca and one professor from the University of California, Davis in a total of eight sessions, four recorded lectures and four online workshops using a video conference platform. After training, primary care teams will implement a collaborative treatment model with a case manager, an individual treatment plan, a follow-up using a combination of validated self-report and clinician-administered instruments, with continuous monthly group supervision by a psychiatrist. All patients enrolled in the clinics assigned to the intervention arm will be treated by the same intervention type according to the CMAD model of treatment. In this arm, patients will receive the usual pharmacological and behavioral treatment, but the health care team will impart the treatment according to the CMAD management model.

### Control arm

The control arm will include 27 hours of training for primary care teams consisting of at least one physician, a psychologist and a social worker on the current clinical guidelines for MD. This guideline outlines a staging algorithm according to severity for the treatment of MD. The training will be conducted by two professors from Universidad de Talca and take place in 5-recorded lectures and three online workshops. After the training, the primary care teams will treat the patients according to the usual treatment. Teams are expected to be familiar with the guidelines and apply them. However, as is the usual treatment, no formal implementation will be offered. All patients enrolled in the clinics assigned to the control arm will be treated according to the usual care. In this arm, patients will receive the usual pharmacological and behavioral treatment, and the health care team will impart the treatment according to the usual clinic management.

### Modifications

For a given trial participant, the assigned study intervention may need to be modified or discontinued by treatment teams for various reasons, including change in diagnosis, risk of suicidality, or withdrawal of participant consent. There will be no strategies to modify the patient’s adherence to treatment as part of this protocol.

### Concomitant care

Primary care teams will provide patients with the pharmacological and behavioral care that are mandatory in the primary health system for patients with MD.

### Outcomes measures

***Primary outcome***

*Change in depressive symptoms*

Difference in depressive symptoms between the two arms of the study will be assessed at baseline and three and six months, as shown in the participants timeline ( Table 1), using the Patient Health Questionnaire validated in Chile. ^21^ This questionnaire is a 9-item self-report survey that uses a 4-point Likert scale to screen for the presence of MD and monitor treatment effects. The total score ranges from 0 to 27, and a greater score means a greater severity of depression. The presence of five or more symptoms in at more than half the days in the last two weeks suggests MD.

**Table 1.** Timeline of health care teams and patients’ measurements and actions as specified in the protocol.

*Time*	Study						
	Teams			Patients			
	*Pre*	*Training*	*Post*	*Enroll*	*Study*		
					0	3 m	6 m
**Intervention**		X					
**Enrollment**				X			
Informed Consent	X			X			
Eligibility				X			
**Evaluations**							
PHQ					X	X	X
GAD-7					X	X	X
OQ-45					X	X	X
ERS					X	X	X
Adherence Scale						X	X
GSES	X		X				

Abbreviations: PHQ, Patient Health Questionnaire; GAD-7, General Anxiety Disorder Scale-7; OQ-45, Outcome questionnaire 45; ERS, Emotion regulation scale; GSES, General self-efficacy scale.

***Secondary outcomes***

*Change in anxiety symptoms*

Difference in anxiety symptoms between the two arms of the study will be evaluated at baseline and three and six months ( Table 1) by the Spanish validated version of the generalized anxiety disorder scale-7 (GAD-7). ^22^ The GAD-7 is a 7-item self-report survey that uses a 3-point Likert scale to screen for generalized anxiety disorder quantify symptom severity. The total score ranges from 0 to 21 points, and greater scores mean greater severity of anxiety.

*Change in interpersonal dysfunction and social role difficulties*

Difference in functional impairment in interpersonal and social areas between the two arms of the study will be evaluated at baseline, at three and at six months ( Table 1) by the subscales of the interpersonal functioning, and social role included in the Outcome Questionnaire 45 (OQ-45). ^23^ The OQ-45 has a 45-item self-report survey that uses a 4-point Likert scale originally developed to monitor patient progress during therapy and after termination. The OQ-45 comprises the subscales of symptom distress, interpersonal relations, and social role. The interpersonal relations subscale includes 12 items addressing loneliness, conflict with others, and marriage and family difficulties, with scores that range from 0 to 48. The social scale consists of nine items tapping into difficulties in the workplace, school or home duties, with scores that range from 0 to 36 points. Greater scores mean worse interpersonal and social functioning.

*Change in emotion regulation*

Difference in the difficulties in emotion regulation between the two arms of the study will be evaluated at baseline, three and six months ( Table 1) by the emotion regulation scale (ERS) validated in Spanish with cutoff scores for the Chilean population. ^24^ ERS contains five subscales addressing emotional rejection, dysregulation, interference, inattention, and confusion. This scale is a 28-item self-report survey that uses a 5-point Likert scale. The scores range from 0 to 140 points, and greater scores indicate increased difficulties with emotional regulation.

*Therapeutic adherence*

Difference in the adherence to treatment between the two arms of the study will be evaluated at three and six months ( Table 1) by the therapeutic adherence scale, ^25^ a 21-item self-reported survey to measure the percentage that patients assign the effectiveness of each behavior. A score of 100 represents the highest adherence possible.

***Other outcomes***

*Self-efficacy of the primary care teams*

The self-efficacy of the primary care team, in implementing the study model, will be evaluated at baseline and after training ( Table 1) by the general self-efficacy scale (GSES) validated in Chile. ^26^ The GSES is a 10-item self-report survey that uses a 7-point Likert scale to measure self-efficacy. Thus, greater scores mean greater self-efficacy.

### Participant timeline

### Sample size

The estimation of the sample size considered a difference of 20% according to previous studies carried out in Chile, ^27^^–^^29^ based in a unilateral model, with an alpha of 5%, a power of 80%, a confidence level of 95% and a maximum variance of 50% resulted in 341 patients. Based on a previous study that determined the clinical and psychosocial variables associated with different evolutions in eight primary care clinics in the Maule region, ^11^ which showed retention of 85%, we adjusted the sample size to 394 patients. Based on a previous study we defined a group of at least 12 primary care clinics in the Maule Region, to be able to access the flow of adult patients with MD required for the feasibility of the study. ^11^

### Recruitment

First, we will enroll the health care clinics through invitation to participate will be made to the health administration of the Maule region. Subsequently, those primary care clinics (Centro de Salud Familiar, in Spanish) authorized by their director will be invited to participate. A minimum of 12 primary care clinics will be enrolled, each with one team composed of a physician, a psychologist and a social worker.

The average socioeconomic level, adult population and patient’s income from 2019 will be used to match pairs of clinics. Later, the clinics will be assigned to the intervention or control groups using a pseudo-random algorithm (randi(2, [1 12], generates a 12-by-1 matrix of random integers between 1 and 2) in Matlab (MathWorks, Natick, MA, USA, RRID:SCR_001622). 

A different researcher, not part of the training team, will make the list of pairs of clinics with similar characteristics in excel and assign each clinic to a number 1 or 2. Then, another researcher will assign the clinics corresponding to the randomization list (sequence of 1 and 2 numbers) to the intervention group and the remaining clinics of each pair to the control group. Finally, this last researcher will define a number code to the intervention and control groups, and a different group of researchers which, blinded to the arm assignments, will perform the data collection and analysis using the number code.

After the training phase, there will be a four month implementation of the CMAD approach in the clinic teams. After the implementation, patients who enter treatment for depression in their respective primary care clinic will be informed of the study and invited to participate by one person of each primary care team. Those who agree to participate, will be asked for their telephone number and researchers blind to the study arms will contact them, providing the informed consent form and then the questionnaires. An alphanumeric code will be assigned for each intervention arm, clinic and patient. Therefore, participants, outcome assessors, and data analysts will be blinded to the intervention allocation.

Patient recruitment is expected to begin on March 1, 2022. To achieve an adequate participant enrolment, each clinic will have a person in charge of inviting all the patients that meet the criteria for enrollment.

### Data collection, management and monitoring

The study will comply with all local research governance requirements for human data collection. A researcher team of psychiatry residents, blind to the intervention arms, will evaluate the participants at the beginning, at three and at six months using a set of instruments. The evaluation team will receive training for data gathering and standardization. The data from different instruments will be entered into a virtual worksheet located on SurveyMonkey by means of a tablet. Each evaluator will enter the data using a password, leaving no information on the devices. The data will be checked for correct entry every day and will be downloaded to the PI personal computer with a password located in an office with key. All data will be entered with the alphanumeric code, which prevents participant de-identification. The data from the participants that are referred to the specialist level will be eliminated from the study.

The data monitoring will be performed by the research team in charge of data collection and will meet every two weeks to evaluate the trial progression and data collection. A different research team in charge of data analysis will perform a checking of the data collected and perform preliminary data analysis to evaluate the progression of the data collection.

### Monitoring

Patients will first be monitored by their respective primary care teams as part of their treatment monthly. In high risk situations such as serious risk of suicide, psychosis and adverse effects of the medications are detected during the evaluations by the researchers (residents), the residents will inform to the researchers in charge of data collection, who in turn will inform the primary care teams. These patients will be excluded from the study as well as patients who do not want to continue with the evaluations.

We have not included plans for collecting, assessing, reporting, and managing solicited and spontaneously reported adverse events and other unintended effects of trial interventions as the medical treatment will not be modified from the national guidelines for MD. The interventions only modify the administration of the clinics’ treatment teams.

### Data analysis

An initial analysis of the sociodemographic and clinical characteristics of the sample will be carried out and the scores of the scales will be calculated. The results will be presented according to the Consolidated Standards of Reporting Trials guide for RCT, with its extensions to cluster interventions. Intention-to-treat analysis of the primary outcome will be performed. In the initial descriptive analysis, the differences in the characteristics of the clinics between the intervention groups will be estimated.

For the analysis of the primary outcome, linear multivariable regression will be used to establish differences at 3 and 6 months, adjusting the results according to the initial data in case of imbalance. In addition, a sensitivity analysis based on different assumptions will also be carried out to evaluate the possible effects of the lack of data. This analysis will also be done for secondary outcomes. Data analysis will be performed with SPSS software (IBM, Armonk, NY, United States, RRID:SCR_002865).

### Ethics approval and consent to participate

Ethical approval for this study was obtained from the institutional Ethics Committee (Comité Ético Científico, Universidad de Talca, protocol # 26-2020). The informed consent document will be available in a Mendeley Data repository. ^34^ Furthermore, informed written consent will be obtained from the participants (mental health team and patients). In case of protocol modifications (for example in the analyses or outcomes) the principal investigator should ask for an amendment to the original ethics informed consent to the Ethics Committee, and the changes should be communicated to all the study team.

The informed consent process will be performed by the research team in charge of the data collection. This team, made up of seven psychiatry residents, will obtained the informed consent in the health care clinic were the participant is admitted in a paper copy that will not have any information about the intervention assignment. During the informed consent procedure, an alphanumeric code will be assigned to the patient, and all the study information will be available under this code but it will not include any personal information that could help to obtain the identity of the participant. One researcher will have the list with the patient’s name and the assigned code in its personal computer secured with a password.

The final data set will be in the computer of the PI, and the researchers in charge of the analyses will have access to the data in the coded form. In case that the results of the study show significantly better outcomes in the intervention arm compared to the control arm, the primary care teams of the control arm will be trained in the CMAD approach.

### Dissemination 

The principal investigator will communicate trial results to participants and healthcare professionals through workshops in the different clinics. The communication of the results to the general public, and other relevant groups will be performed trough seminars in the University of Talca. Finally, the communication of the results to the medical and scientific community will be done through the publication in specialized journals and the data sharing of the results in databases (eg, Mendeley). Moreover, the protocol will be available after the study has ended. Regarding the authorship legibility for the protocol, we will include all researchers that contributed significantly to the design, implementation the clinics training, data collection and analysis.

### Study status

This study has concluded the health clinics’ recruitment as well as the health care team’s informed consent and training in both study arms. Patient recruitment is expected to begin on March 1, 2022.

## Discussion

Collaborative models have proven to be the most effective to improve the outcomes for the treatment of MD in primary care. ^18^ However, in Chile, a single study that applied this model on the current government clinical guideline's recommendations showed no significant efficacy in the resolution of depressive symptoms. ^28^ As far as we know, there is no evidence on the efficacy of a collaborative model on the complex, difficult-to-treat depression subtype, which includes the recognition by primary care teams of clinical and functional variables associated with exposure to adversity and trauma. ^13^^,^^29^^,^^30^

This project seeks to test a clinical approach that includes a comprehensive, multidisciplinary clinical evaluation, and promotes the synergy of the entire health team, so that patients with difficult-to-treat depression, with neurobiological and psychological vulnerabilities associated with a history of biographical adversity, receive consistent treatment over time. It also entails close collaboration between the primary and secondary health system levels, which may reduce emotional distress in the treating professionals themselves.

Among the limitations, it should be noted that the continued education intervention will recruit team members who wish to participate. The sample of health care professionals may not be representative of health care professionals working in primary care in Chile. Professionals who are especially motivated in their continued education may be overrepresented among those who consent to participate. In addition, it is possible that this group of providers may have a higher-than-average competency level in some of the skills and attitudes fostered by the proposed approach.

As an additional limitation, results from this randomized trial involving many low income, rural and semi-rural patients in central Chile may not be extended to other more socioeconomically advantaged populations or patients living elsewhere in the country. Surveys using nationally representative samples have found up to 15-fold differences in prevalence between richer and poorer regions even if at the country level prevalence of MD has not changed significantly in the period of observation. ^31^ Furthermore, Chile’s income inequality gap is also more than 65% wider than the average in a group of mostly rich countries, ^32^ and meta-analysis data demonstrate greater risk of MD in populations with higher income inequality relative to populations with lower inequality. ^33^ Overall, the focus on primary care in the Maule Region of Chile seems justified on population health and social justice considerations. We hope that results from this trial will help address the existing low remission rates of MD in primary care, especially in patients presenting with difficult-to-treat, complex clinical presentations.

## Consent for publication

Not applicable.

## Data availability

### Underlying data

No underlying data are associated with this article.

### Extended data

Mendeley Data: Collaborative multidimensional approach to improve the resolution of depression in primary care in Chile https://doi.org/10.17632/jdftfbpvkn.1 ^34^

This project contains the following extended data:

- InformedConsent
  o Acta de aprobación V. Vitriol.pdf (Minutes of the Institutional Ethic Committee reporting the approval of the Informed Consent documents)
  o Consentimiento informado aprobado CEC.pdf (Informed consent document for participants)
  o Consentimiento informado profesional aprobado CEC.pdf (Informed consent document for primary care teams)
- Primary Care Clinic Lists
  o Primary Care clinics.pdf (Clinics selected for trial).
- Project Acceptance Letter
  o Carta N°988 Adj. XVII Fonis U. de Talca.pdf (Allocation of Funds for the study)

### Reporting guidelines

Mendeley Data: Collaborative multidimensional approach to improve the resolution of depression in primary care in Chile https://doi.org/10.17632/jdftfbpvkn.1 ^34^

This project contains the following reporting guidelines:

- SPIRIT-Checklist-31 Dec 2021.pdf

Data are available under the terms of the Creative Commons Attribution 4.0 International license (CC-BY 4.0).

## Roles and responsibilities

The research team responsible for this study belong to the School of Medicine of the University of Talca and the University of Davis, in collaboration with the Maule Health Service.

Principal Investigator: Verónica Vitriol

Study design: Verónica Vitriol, Alfredo Cancino, Andrés Sciolla and Maria de la Luz Aylwin

Clinic recruitment: Marcela Ormazábal, Sergio Guiñez

Clinic Team Data collection: Sergio Guiñez, Johanna Kreither

Pairing and assignment: Marcela Ormazábal, Maria de La Luz Aylwin

Training Primary Care Teams CMAD: Veronica Vitriol, Alfredo Cancino, Andres Sciolla

Training Primary Care Teams Depression Clinical Guideline: Jorge Calvo

Patient recruitment: Marcela Ormazábal, Soledad Ballesteros

Patient data collection and monitoring: Marcela Ormazábal, Soledad Ballesteros

Data analysis and interpretation: Veronica Vitriol, Alfredo Cancino, Andres Sciolla and Johanna Kreither

Research report writing: Veronica Vitriol, Alfredo Cancino, Andres Sciolla, Maria de la Luz Aylwin, Sergio Guiñez, Johanna Kreither, Jorge Calvo

## Authors' information

• Verónica G. Vitriol MD, Escuela de Medicina, Universidad de Talca, Talca, Chile
• Alfredo Cancino MD, Escuela de Medicina, Universidad de Talca, Talca, Chile
• Andrés Sciolla MD, Department of Psychiatry, Medical School, UC Davis, USA
• Sergio Guiñez PhD, Escuela de Medicina, Universidad de Talca, Talca, Chile
• Jorge Calvo MD, Escuela de Medicina, Universidad de Talca, Talca, Chile
• Marcela Ormazábal Ms, Departamento APS, Programas y Ciclo Vital, Subdirección de Gestión Asistencial, Dirección Servicio de Salud del Maule, Talca, Chile
• Johanna Kreither PhD, Centro de Investigación en Ciencias Cognitivas, Centro de Psicología Aplicada Facultad de Psicología, Universidad Talca, Talca Chile
• Soledad Ballesteros MD, Escuela de Medicina, Universidad de Talca, Talca, Chile
• María de la Luz Aylwin PhD, Centro de Investigaciones Médicas, Escuela de Medicina, PIA Ciencias Cognitivas, Centro de Investigación en Ciencias Cognitivas, Universidad de Talca, Talca, Chile
