# Effectiveness of a multidimensional collaborative approach versus usual care in the treatment of adult depression in primary care in Chile: study protocol for a single blinded cluster randomized controlled trial

**DOI:** 10.12688/f1000research.75764.2

**Published:** 2024-10-11

**Authors:** VERONICA VITRIOL, ALFREDO CANCINO, ANDRES SCIOLLA, SERGIO GUIÑEZ, JORGE CALVO, MARCELA ORMAZABAL, JOHANNA KREITHER, SOLEDAD BALLESTEROS, MARIA DE LA LUZ AYLWIN

**Affiliations:** 1 Escuela de Medicina, Universidad de Talca, Talca, Maule, 3460000, Chile; 2 Department of Psychiatry and Behavioral Sciences School of Medicine, UC Davis Medical Center, Sacramento, Californa, 95616, USA; 3 Departamento Atención Primaria, Servicio de Salud del Maule,, Talca, Maule, 3460000, Chile; 4 Centro de Psicología Aplicada, Universidad Talca, Talca, Maule, 3600000, Chile; 5 Centro de Investigación en Ciencias Cognitivas, Universidad de Talca, Talca, Maule, 360000, Chile; 6 Centro de Investigaciones Médicas, Universidad de Talca, Talca, Maule, 3600000, Chile

**Keywords:** Depression treatment, primary care, collaborative care, trauma-informed care, randomized controlled trial

## Abstract

**Background:**

Major depression (MD) is a prevalent and disabling condition in Chile, with most cases being treated at the primary care level. In Chilean primary care, the authors have identified key factors associated with more complex presentations of MD and a poorer prognosis, such as a history of childhood trauma, suicidality, and comorbidities. These findings underscore the need for a multidimensional, trauma-informed, and interprofessional approach to the treatment of depression.

**Methods:**

This protocol is a two-arm, single-blinded, cluster RCT to compare the effectiveness of a collaborative multidimensional approach for depression (CMAD) versus usual care to treat MD in primary care clinics in Chile. In total, 394 depressed adults from 18 to 65 years of age in twelve clinics located in Chile’s Maule Region will be consented to participate in the study. Patients and care teams from each clinic will be randomized to the intervention or to the control arm.

Interprofessional teams in the intervention arm will attend 27 hours of didactic and active learning sessions focused on clinical competences to effectively engage, treat and follow up patients with the factors associated to the complex presentation of MD. Team in the control arm will receive 27 didactic sessions on current clinical guidelines for MD.

Patients of both arms will be blindly assessed at baseline, three months, and six months. The primary outcome will be the reduction in depressive symptoms, with secondary outcomes including improvements in anxiety symptoms, interpersonal and social functioning, and treatment adherence.

**Discussion:**

This protocol proposes the evaluation of an intervention designed to improve depression symptoms by enhancing the clinical competencies of primary care teams. These competencies are structured around collaborative care and trauma-informed practices.

**Trial registration:**

NCT05016388, registered on 16 August 2021 at ClinicalTrials.gov.

**Abbreviations**
CMAD: Collaborative multidimensional approach for depression
GAD-7: General Anxiety Disorder Scale-7
GSES: General self-efficacy scale
ERS: Emotion regulation scale
MD: Major depression
MINI: Mini-International Neuro-psychiatric Interview
PHQ9: Patient Health Questionnaire
OQ-45: Outcome Questionnaire 45
RCT: Randomized controlled trial
UC: Usual Care

## Background

Depression is the leading cause of disability worldwide, contributing significantly to the overall global burden of morbidity and mortality. ^1^ It is estimated that it affects almost 280 million people worldwide. ^1^ In Chile, depression constitutes a significant public health problem. ^2^ Findings before the SARS-CoV-2 pandemic, showed that 18.2% of the adult Chilean population reported depressive symptoms, and 6.2% met the criteria for major depression (MD). ^3^^,^^4^ This prevalence increases with the pandemic. ^5^ Furthermore, it affects twice as many women as men, is the second leading cause of disability-adjusted life years and the first among women between 20 and 44 years. ^2^

Since 2001 there has been a Chilean national mental health program to treat MD. From 2006, this program has been known as Explicit Health Guarantees (Garantías Explícitas en Salud, GES, in Spanish), and is regulated by ministerial clinical guideline (Guía Clínica, 2013). ^6^ According to existing data, primary care treat 90% of MD cases. ^7^ Only those with current suicide attempts, suspected bipolarity or psychosis are referred to the specialty level. ^2^^,^^7^

Despite the implementation of the governmental guidelines, evidence shows limited competence in primary care teams to address MD, ^7^ with remission rates close to 50% at one year of follow-up. ^8^^,^^9^ This lack of remission is associated with a high prevalence of comorbid anxiety disorders, adverse childhood experiences, history of suicidality, and interpersonal difficulties. ^10^^–^^12^ These characteristics, which have been reported in refractory depression, ^13^ can be understood in light of the psychological and neurobiological sequel of exposure to adverse biographical experiences. ^14^ Currently, depression treatment guidelines lack specific recommendations to address these issues. ^6^

According to a consensus of international experts, the clinical characteristics of patients with refractory depression are better conceptualized as difficult-to-treat depression. ^13^ This conceptualization implies an understanding of depression as a chronic disease, which requires a collaborative, multidimensional, biopsychosocial clinical approach and an orientation to the management of symptoms and functional recovery. ^13^ Working with similar populations, others have proposed the adoption of trauma-informed care principles and approaches, considering the cumulative interactions between trauma exposure, socioeconomic disadvantage, depression and suicidality. ^15^^,^^16^

Both difficult-to-treat depression and trauma-informed care imply a collaborative organization health model similar to that proposed by Wagner *et al* in the management of chronic diseases, ^17^ including anxiety and depressive disorders as proposed by Archer *et al.* ^18^ It involves the presence of a case manager, a structured patient-centered approach, scheduled follow-up visits, the use of validated measures to monitor treatment response, and interprofessional communication between the different levels of care. ^18^

Considering all of the above, we hypothesize that a continuing education program for primary care teams that focuses on a bio-psychosocial, trauma-informed care approach implemented in a collaborative model will improve the outcomes of depression in comparison to usual care. Specifically, we hypothesize that the collaborative multidimensional approach for depression (CMAD) in primary care will demonstrate greater efficacy relative to usual care (UC) based on the clinical guidelines, considering:

1. Adult depressed patients in the CMAD arm significantly reduce depressive symptoms compared to the control group at 3 and 6 months after admission.
2. Adult depressed patients in the CMAD arm significantly reduce anxiety symptoms compared to the control group at 3 and 6 months after admission.
3. Adult depressed patients in the CMAD arm significantly reduce interpersonal dysfunction, social impairment, and emotional dysregulation compared to the control group at 3 and 6 months after admission
4. Adult depressed patients in the CMAD arm achieve a significantly greater adherence than the control group at 3 and 6 months after admission.

The general aim of this study is to compare the effectiveness of a CMAD for MD versus UC on clinical and functional outcomes in a cluster randomized clinical trial (RCT) conducted in primary care clinics in the Maule Region of Chile.

The specific aims of this study are:

1. To compare changes in depressive symptoms between baseline and follow-up in adults treated for MD in primary care Maule clinics, using CMAD versus UC
2. To compare changes in anxiety symptoms between baseline and follow-up in adult depressed patients treated for MD in primary care Maule clinics, using CMAD versus UC.
3. To compare changes in symptoms of emotional regulation, interpersonal and social functions between baseline and follow-up in adult depressive patients treated for MD in primary care Maule clinics, using CMAD versus UC.
4. To compare changes in therapeutic adherence between three and six months in adult depressive patients treated for MD in primary care Maule clinics, using CMAD versus UC.

## Methods

### Design

This study protocol is a cluster RCT with two parallel arms and single-blind outcome evaluation.

### Study setting

The Chilean healthcare system is divided into two main sectors: public and private. The public sector, managed by the National Health Fund (FONASA), covers 75% of the population, particularly those with lower incomes. Public healthcare services are provided through a network of health facilities, including hospitals and primary care centers. Primary care centers are a fundamental part of the Chilean public healthcare system and are primarily managed at the municipal level. Family health centers provide basic and preventive care, and services are free for patients.

To meet the objectives of this protocol, primary care clinics in the Maule Region, located in the municipalities of Talca, Curicó, Constitución, Sagrada Familia, Romeral, and Pelarco, are invited to participate. These centers represent both urban and rural areas in a disadvantaged region, where socioeconomic and educational levels are particularly low. ^19^ To obtain the sample, 12 centers are identified, and matched based on according to similar average socioeconomic and educational characteristics. Additionaly, the selection ensures that there is no cross-contamination between the patients in the control clinics and the intervention clinics.

The list of primary care clinics participating can be found at doi: 10.17632/jdftfbpvkn.1.

### Eligibility criteria

Adults between 18 and 65 years who enter treatment for depression in primary care clinics of the Maule Region are invited to participate in an evaluation after they sign a written informed consent statement. ^34^ Those with a confirmed diagnosis of MD, according to the Mini-international Neuro-psychiatric interview (MINI), are included in the study. ^20^ Exclusion criteria include the inability or unwillingness to sign informed consent, sensory disabilities, lack of access to a telephone, or referral to a specialist for conditions such as current suicidal attempts, bipolar disorder, or severe drug addiction.

### Sample size

The sample size estimation account for a 20% difference, as suggested by previous studies conducted in Chile. ^21^^,^^22^ This calculation is based on a one-sided model with an alpha of 5%, a power of 80%, a confidence level of 95%, and a maximum variance of 50%. A total of 240 depressed individuals are required, with 120 participants in each group (intervention and control). Bases on previous data, the intraclass correlation coefficient (ICC) is estimated to be 0.0337, ^22^ resulting in a design effect of 1.64 due to the involvement of 12 clinics. Applying this design effect increases the sample size to 341, based on a prior study the sample size is set at 394 patients. ^8^

### Recruitment and randomization

Twelve eligible primary care clinics, each with a team consisting of a physician, psychologist, and social worker, are enrolled to obtain the sample. The clinics are invited through an official request to the Maule Region health administration. Once authorized by their directors, the selected primary care clinics (Centros de Salud Familiar) participate. Clinics are matched based on similar average socioeconomic and educational characteristics, ensuring no cross-contamination between patients from control and intervention clinics. Data from 2019 on socioeconomic level, adult population, and patient income are used to match clinic pairs. ^19^

A separate researcher, not part of the training team, create a list of clinic pairs, with similar characteristics in Excel and assign each clinic a number (1 or 2). Clinics are then assigned to either the intervention or control group using a pseudo-random algorithm (randi(2, [1 12]), which generates a 12-by-1 matrix of random integers between 1 and 2) in Matlab (MathWorks, Natick, MA, USA, RRID).

Finally, this researcher assigns number codes to the intervention and control groups. A different group of researchers, blinded to the group assignments, performs data collection and analysis using these codes. Patients who begin treatment for depression at their respective primary care clinics are informed of the study and invited to participate by a designated member of the primary care team. Those who agree to participate provide their telephone numbers, and researchers, blinded to group assignments, contact them to obtain informed consent and administer the questionnaires. An alphanumeric code is assigned to each intervention arm, clinic, and patient. Consequently, participants, outcome assessors, and data analysts remain blinded to the intervention allocation.

Patient recruitment begin on March 1, 2022 and conclude by November 2022.

### Interventions

***Intervention arm***

The intervention arm consists of training in the CMAD approach and its implementation.

***Training***

The training consists of 27 hours of lectures for a team that includes at least one physician, one psychologist, and one social worker. The CMAD approach integrates knowledge, attitudes, and skills for managing clinical manifestations associated with adversity or trauma across the lifespan. The training covers topics such as the general framework, trauma-informed care, bonding and mentalization, resilience, as well as four workshops focused on interprofessional diagnosis, treatment, follow-up, and collaborative work. These competencies complement, rather than replace, the diagnostic and treatment recommendations outlined in the current clinical guidelines for depression.

The training is delivered by two professors from Universidad de Talca (VV, AC) and one professor from the University of California, Davis (ASc).

***Implementation***

The CMAD follows an integrated interprofessional model of patient care, which includes assigning a case manager, developing individualized treatment plans, and conducting follow-ups using validated self-report tools. Continuous supervision from a specialist is provided through consultations for at least six months. The case manager promotes collaboration among professionals, including psychiatric care, to ensure comprehensive treatment.

At the start, all patients undergo a multidimensional, interprofessional interview that assesses categorical diagnoses, clinical severity, screens for bipolar disorder, diagnoses medical and psychiatric comorbidities, and explores the patient’s psychobiographical history to better understand the reason for consultation. Following the interview, the team collaborates to establish a treatment goal aimed at addressing the patient’s immediate concerns within a crisis intervention framework.

The team monitors patient progress and well-being using self-report instruments such as the PHQ-9 and GAD-7. ^23^^,^^24^ Monthly evaluations are conducted by a specialist in collaboration with the care team and the patient through teleconsultation sessions. In this arm, patients receive the usual pharmacological treatment.

### Control arm

The control arm includes 27 hours of training for primary care teams consisting of at least one physician, a psychologist, and a social worker on the current clinical guidelines for MD. This guideline outlines a staging algorithm according to severity for the treatment of MD. The training is conducted by two professors from Universidad de Talca and takes place in 5 recorded lectures and three online workshops. After the training, the primary care teams treat the patients according to the usual treatment. Teams are expected to be familiar with the guidelines and apply them. However, as is usual, no formal implementation is offered. In this arm, patients receive the usual pharmacological and behavioral treatment, and the health care team imparts the treatment according to the usual clinic management.

***Blinding and Follow-up***

At each clinic, a member of the primary care team recruits study participants who provide written informed consent. These participants are then referred to two psychologists (MO, SB), who remain unaware of the patients’ assigned groups and allocate them to an external evaluation team composed of ten psychiatry residents. A protocol for managing emergency cases is in place.

The residents receive a 4-hour training session on data standardization and are supervised by MO and SB. The participants are evaluated at the time of inclusion using a semi-structured clinical interview, the ACEs inventory, the MINI, and a set of instruments to assess outcomes at baseline, three months, and six months.

### Participant timeline

**Table 1.** Timeline of health care teams and patients’ measurements and actions as specified in the protocol.

Time	Study						
	Teams			Patients			
	Pre	Training	Post	Enroll	Study		
					0	3 m	6 m
**Intervention**		X					
**Enrollment**				X			
*Informed Consent*				X			
*Eligibility*				X			
**Evaluations**							
*PHQ*					X	X	X
*GAD-7*					X	X	X
*OQ-45*					X	X	X
*ERS*					X	X	X
*Adherence Scale*						X	X
*GSES*	X		X				

Abbreviations: PHQ, Patient Health Questionnaire; GAD-7, General Anxiety Disorder Scale-7; OQ-45, Outcome questionnaire 45; ERS, Emotion regulation scale; GSES, General self-efficacy scale.

### Outcomes measures

***Primary outcome***

*Change in depressive symptoms*

Differences in depressive symptoms between baseline and follow-up across the two study arms are assessed at baseline, three months, and six months, as shown in the participants’ timeline ( Table 1), using the Patient Health Questionnaire validated in Chile. This questionnaire is a 9-item self-report survey that uses a 4-point Likert scale to screen for the presence of MD and monitor treatment effects. The total score ranges from 0 to 27, with higher scores indicating greater severity of depression. The presence of five or more symptoms on more than half of the days in the past two weeks suggests major depresson ( Table 1), using the Patient Health Questionnaire validated in Chile. ^23^ This questionnaire is a 9-item self-report survey that uses a 4-point Likert scale to screen for the presence of MD and monitor treatment effects. The total score ranges from 0 to 27, and a greater score means a greater severity of depression. The presence of five or more symptoms in at more than half the days in the last two weeks suggests MD.

***Secondary outcomes***

*Change in anxiety symptoms*

Difference in anxiety symptoms between baseline and follow up across the two arms of the study will be assessed at baseline and three and six months ( Table 1) by the Spanish validated version of the generalized anxiety disorder scale-7 (GAD-7). ^24^ The GAD-7 is a 7-item self-report survey that uses a 3-point Likert scale to screen for generalized anxiety disorder quantify symptom severity. The total score ranges from 0 to 21 points, and greater scores mean greater severity of anxiety.

*Change in interpersonal dysfunction and social role difficulties*

Difference in functional impairment in interpersonal and social areas between baseline and follow-up across the two arms of the study will be assessed at baseline, at three and at six months ( Table 1) by the subscales of the interpersonal functioning, and social role included in the Outcome Questionnaire 45 (OQ-45). ^25^ The OQ-45 has a 45-item self-report survey that uses a 4-point Likert scale originally developed to monitor patient progress during therapy and after termination. The OQ-45 comprises the subscales of symptom distress, interpersonal relations, and social role. The interpersonal relations subscale includes 12 items addressing loneliness, conflict with others, and marriage and family difficulties, with scores that range from 0 to 48. The social scale consists of nine items tapping into difficulties in the workplace, school or home duties, with scores that range from 0 to 36 points. Greater scores mean worse interpersonal and social functioning.

*Change in emotion regulation*

Difference in the difficulties in emotion regulation between baseline and follow-up across the two arms of the study will be assesed at baseline, three and six months ( Table 1) by the emotion regulation scale (ERS) validated in Spanish with cutoff scores for the Chilean population. ^26^ ERS contains five subscales addressing emotional rejection, dysregulation, interference, inattention, and confusion. This scale is a 28-item self-report survey that uses a 5-point Likert scale. The scores range from 0 to 140 points, and greater scores indicate increased difficulties with emotional regulation.

*Therapeutic adherence*

Difference in the adherence to treatment between the two arms of the study will be assessed at three and six months ( Table 1) by the therapeutic adherence scale, ^27^ a 21-item self-reported survey to measure the percentage that patients assign the effectiveness of each behavior. A score of 100 represents the highest adherence possible.

***Other outcomes***

*Self-efficacy of the primary care teams*

The self-efficacy of the primary care team, in implementing the study model, will be evaluated at baseline and after training ( Table 1) by the general self-efficacy scale (GSES) validated in Chile. ^28^ The GSES is a 10-item self-report survey that uses a 7-point Likert scale to measure self-efficacy. Thus, greater scores mean greater self-efficacy.

### Data collection, management and monitoring

The study complies with all local research governance requirements for human data collection. The data from different instruments are entered into a virtual worksheet located on a secure website ( https://www.surveymonkey.com/) using a tablet. Each evaluator enters the data using a password, leaving no information on the devices. The data are checked for correct entry every day and downloaded to the PI’s personal computer, which is password-protected and located in a locked office. All data are entered with an alphanumeric code to prevent participant identification.

The data monitoring is performed by the research team in charge of data collection, which meets every two weeks to evaluate the trial progression and data collection. A different research team, responsible for data analysis, checks the collected data and performs preliminary data analysis to assess the progression of data collection.

### Monitoring

Patients are first monitored by their respective primary care teams as part of their treatment. If risk situations such as serious suicide risk, psychosis, or adverse effects of medications are detected during evaluations by the residents, they notify the researchers responsible for data collection, who, in turn, inform the primary care teams. The healthcare teams then take steps to safeguard the patient’s well-being in accordance with current recommendations.

We do not include plans for collecting, assessing, reporting, and managing solicited and spontaneously reported adverse events and other unintended effects of trial interventions, as pharmachological treatment is not modified.

### Data analysis

The results are presented following the Consolidated Standards of Reporting Trials (CONSORT) guidelines for randomized controlled trials (RCTs), including extensions for cluster interventions. An initial analysis of the sociodemographic and clinical characteristics of the sample is conducted, and scale scores are calculated.

For the sensitivity analysis, missing data are addressed using the Last Observation Carried Forward (LOCF) imputation method. Additionally, sensitivity analyses are conducted under different assumptions to assess the potential impact of missing data. For both primary and secondary outcome analyses, multivariable linear regression is used to assess differences at 3 and 6 months, with adjustments made based on baseline data to account for any imbalances. Two key assumptions of the regression analysis are tested: the absence of baseline differences between groups and the homogeneity of regression for baseline and six-month follow-up data.

Before conducting the regression analysis, the intracluster correlation coefficient (ICC) is calculated for each outcome, both at baseline and at the six-month follow-up, except for adherence, which is evaluated at both the three- and six-month follow-ups. The ICC is estimated as the ratio of between-subject variance to total variance (between- plus within-subject variance) derived from the ANOVA. This analysis is also applied to the secondary outcomes.

All statistical analyses are performed using SPSS version 21 and Jamovi (IBM, Armonk, NY, United States, RRID).

### Ethics approval and consent to participate

Ethical approval for this study was obtained from the institutional Ethics Committee (Comité Ético Científico, Universidad de Talca, protocol # 26-2020). The informed consent document is available in a Mendeley Data repository. ^34^ Furthermore, informed written consent is obtained from the participants (mental health team and patients).

The informed consent process is conducted by the research team responsible for data collection. This team, obtains the informed consent at the healthcare clinic where the participant is admitted, using a paper copy that does not contain any information about the intervention assignment. During the informed consent procedure, an alphanumeric code is assigned to each patient, and all study information is linked to this code, without including any personal information that could reveal the participant’s identity. One researcher maintains a list of the patient’s names and their assigned codes, securely stored on a password-protected personal computer.

The final data set is stored on the PI’s computer, and the researchers responsible for the analysis have access to the data in its coded form. If the study results show significantly better outcomes in the intervention arm compared to the control arm, the primary care teams in the control arm are trained in the CMAD approach.

### Dissemination

The investigators and sponsor communicate the trial results to participants and healthcare professionals through workshops held at various clinics. The communication of results to the general public and other relevant groups is conducted via seminars at the University of Talca. Additionally, the dissemination of results to the medical and scientific community takes place through publication in specialized journals and by sharing the data in databases (e.g., https://data.mendeley.com/). The full protocol is shared after the study has concluded.

### Study status

The study has concluded.

## Discussion

Collaborative models have proven to be the most effective to improve the outcomes for the treatment of MD in primary care. ^18^ However, in Chile, a single study that applied this model on the current government clinical guideline’s recommendations showed no significant efficacy in the resolution of depressive symptoms. ^29^ As far as we know, there is no evidence on the efficacy of a collaborative model on the complex, difficult-to-treat depression subtype, which includes the recognition by primary care teams of clinical and functional variables associated with exposure to adversity and trauma. ^30^^,^^31^

This project seeks to test a clinical approach that includes a comprehensive, multidisciplinary clinical evaluation, and promotes the synergy of the entire health team, so that patients with difficult-to-treat depression, with neurobiological and psychological vulnerabilities associated with a history of biographical adversity, receive consistent treatment over time. It also entails close collaboration between the primary and secondary health system levels, which may reduce emotional distress in the treating professionals themselves.

Among the limitations, it should be noted that the continued education intervention recruits team members on a voluntary basis. As a result, the sample of health care professionals may not be representative of all primary care professionals in Chile. Those especially motivated in continuing education may be overrepresented among participants. Additionally, this group of providers may possess a higher-than-average competency level in some of the skills and attitudes promoted by the intervention.

As an additional limitation, results from this randomized trial involving many low income, rural and semi-rural patients in central Chile may not be extended to other more socioeconomically advantaged populations or patients living elsewhere in the country. Surveys using nationally representative samples have found up to 15-fold differences in prevalence between richer and poorer regions even if at the country level prevalence of MD has not changed significantly in the period of observation. ^32^ Furthermore, Chile’s income inequality gap is also more than 65% wider than the average in a group of mostly rich countries, ^33^ and meta-analysis data demonstrate greater risk of MD in populations with higher income inequality relative to populations with lower inequality. ^34^ Overall, the focus on primary care in the Maule Region of Chile seems justified on population health and social justice considerations. We hope that results from this trial will help address the existing low remission rates of MD in primary care, especially in patients presenting with difficult-to-treat, complex clinical presentations.

## Consent for publication

Not applicable.

## Data availability

### Underlying data

No underlying data are associated with this article.

### Extended data

Mendeley Data: Collaborative multidimensional approach to improve the resolution of depression in primary care in Chile https://doi.org/10.17632/jdftfbpvkn.1 ^35^

This project contains the following extended data:

• InformedConsent
  ○ Acta de aprobación V. Vitriol.pdf (Minutes of the Institutional Ethic Committee reporting the approval of the Informed Consent documents)
  ○ Consentimiento informado aprobado CEC.pdf (Informed consent document for participants)
  ○ Consentimiento informado profesional aprobado CEC.pdf (Informed consent document for primary care teams)
• Primary Care Clinic Lists
  ○ Primary Care clinics.pdf (Clinics selected for trial).
• Project Acceptance Letter
  ○ Carta N°988 Adj. XVII Fonis U. de Talca.pdf (Allocation of Funds for the study)

### Reporting guidelines

Mendeley Data: Collaborative multidimensional approach to improve the resolution of depression in primary care in Chile https://doi.org/10.17632/jdftfbpvkn.1 ^35^

This project contains the following reporting guidelines:

• SPIRIT-Checklist-31 Dec 2021.pdf

Data are available under the terms of the Creative Commons Attribution 4.0 International license (CC-BY 4.0).

## Roles and responsibilities

The research team responsible for this study belong to the School of Medicine of the University of Talca and the University of Davis, in collaboration with the Maule Health Service.

Principal Investigator: Verónica Vitriol

Study design: Verónica Vitriol, Alfredo Cancino, Andrés Sciolla and Maria de la Luz Aylwin

Clinic recruitment: Marcela Ormazábal, Sergio Guiñez

Clinic Team Data collection: Sergio Guiñez, Johanna Kreither

Pairing and assignment: Marcela Ormazábal, Maria de La Luz Aylwin

Training Primary Care Teams CMAD: Veronica Vitriol, Alfredo Cancino, Andres Sciolla

Training Primary Care Teams Depression Clinical Guideline: Jorge Calvo

Patient recruitment: Marcela Ormazábal, Soledad Ballesteros

Patient data collection and monitoring: Marcela Ormazábal, Soledad Ballesteros

Data analysis and interpretation: Veronica Vitriol, Alfredo Cancino, Andres Sciolla and Johanna Kreither

Research report writing: Veronica Vitriol, Alfredo Cancino, Andres Sciolla, Maria de la Luz Aylwin, Sergio Guiñez, Johanna Kreither, Jorge Calvo

## Authors’ information

• Verónica G. Vitriol MD, Escuela de Medicina, Universidad de Talca, Talca, Chile
• Alfredo Cancino MD, Escuela de Medicina, Universidad de Talca, Talca, Chile
• Andrés Sciolla MD, Department of Psychiatry, Medical School, UC Davis, USA
• Sergio Guiñez PhD, Escuela de Medicina, Universidad de Talca, Talca, Chile
• Jorge Calvo MD, Escuela de Medicina, Universidad de Talca, Talca, Chile
• Marcela Ormazábal Ms, Departamento APS, Programas y Ciclo Vital, Subdirección de Gestión Asistencial, Dirección Servicio de Salud del Maule, Talca, Chile
• Johanna Kreither PhD, Centro de Investigación en Ciencias Cognitivas, Centro de Psicología Aplicada Facultad de Psicología, Universidad Talca, Talca Chile
• Soledad Ballesteros MD, Escuela de Medicina, Universidad de Talca, Talca, Chile
• María de la Luz Aylwin PhD, Centro de Investigaciones Médicas, Escuela de Medicina, PIA Ciencias Cognitivas, Centro de Investigación en Ciencias Cognitivas, Universidad de Talca, Talca, Chile
