# Supplementary material for: Effectiveness of a multidimensional collaborative approach versus usual care in the treatment of adult depression in primary care in Chile: study protocol for a single blinded cluster randomized controlled trial
Source: F1000Res. 2024 Oct 11;11:203. Originally published 2022 Feb 17. [Version 2] doi: 10.12688/f1000research.75764.2 (PMC11502995; doi:10.12688/f1000research.75764.2)
Supplement: Peer review history

# Reviewer report: Reviewer response for version 2

**DOI:** 10.5256/f1000research.172642.r330869

**Published:** 2024-10-24

**Authors:** Lene F. Eplov, Marte Ustrup

**Affiliations:** 1 Copenhagen Research Center for Mental Health - CORE, Mental Health Centre Copenhagen, Copenhagen, Denmark; 2 Copenhagen Research Unit for Recovery, Mental Health Centre Amager, Copenhagen, Denmark

We are very pleased to see that all our reviewer comments have been addressed. We have no further comments to make and approve this final version.

Is the study design appropriate for the research question?

Yes

Is the rationale for, and objectives of, the study clearly described?

Yes

Are sufficient details of the methods provided to allow replication by others?

Partly

Are the datasets clearly presented in a useable and accessible format?

Not applicable

Reviewer Expertise:

psychiatry, mental health, recovery, interventions, implementation, collaborative care

We confirm that we have read this submission and believe that we have an appropriate level of expertise to confirm that it is of an acceptable scientific standard.

# Reviewer report: Reviewer response for version 1

**DOI:** 10.5256/f1000research.79682.r281255

**Published:** 2024-09-12

**Authors:** Lene F. Eplov, Marte Ustrup

**Affiliations:** 1 Copenhagen Research Center for Mental Health - CORE, Mental Health Centre Copenhagen, Copenhagen, Denmark; 2 Copenhagen Research Unit for Recovery, Mental Health Centre Amager, Copenhagen, Denmark

The paper is a study protocol for a cluster randomized controlled trial evaluating the effect of a collaborative multidimensional approach for major depression (MD). This is an important study, as MD constitutes are large burden in Chile and because the research group has previously observed a complex depressive subtype with poor response to standard treatment.

 The rationale for the study is clearly described.

 The choice of a multidimensional, collaborative, and trauma-informed approach is well-justified and seems an appropriate choice, based on the literature and the groups previous studies.

 Overall, the study protocol is clearly structured and conforms with the CONSORT guidelines.

 However, there are some major issues as well as minor issues that needs to be addressed.

**Major issues**

 Abstract:

- The primary outcome is not described in the section “Methods” in the abstract. This is essential information and should be provided here.

Background:

- The section clearly describes the scientific background and rationale. However, the authors use a lot of space to describe a subgroup among people with MD and although this is interesting, the RCT is investigation the effect of the intervention on the whole population with MD in primary care. Furthermore, it doesn’t look like they are going to do a subgroup analysis on the mentioned subgroup and the power calculation is based on wanting an analysis of the effect on the whole group, leading to the fact that there is not enough power to examine the effect of the intervention on the mentioned subgroup. Therefore, the introduction needs to have the main focus on describing the need of the intervention for the whole group of people with MD in primary care.

Methods:

- The section “Modifications” is hard to understand. Is the modification made in the treatment, then move the section up to the description of the “intervention arm”, if it is part of the must conduct the analyses then move the section to “Data analysis”. Be aware then that you can’t write that you will perform intention-to-treat analysis, as described under the section “Data analysis”.
- Sample size: It is unclear if the sample size calculation takes the cluster design of the RCT into account. There is no mentioning of assumptions, such as approximate equal-sized clusters, or intra-class correlation (ICC).
- Data analysis: As for the sample size calculation, it is unclear if and how the analyses take the clustering of individuals in clinics into account.

**Minor issues**

 Abstract:

- The section “Discussion” is more of a conclusion, as if the trial has already been completed and analyzed. Instead, rephrase to e.g., “This trial will provide information on whether…”.
- If the trial has been registered, e.g., at ClinicalTrials.gov, please provide registration number and date at the end of the Abstract.

Hypotheses and aims:

- Instead of listing five aims and four hypotheses, only list those related to the primary outcome, i.e. aim 3. The protocol only describes the RCT and therefore it is not an aim to, for example, design the intervention (aim 1), unless this process is also described in details.
- Hypotheses 1 and 2: Change the wording “improve depressive symptoms” to “a reduction in depressive symptoms”, as this is more specific.
- Aim 3: To make the aim and outcome clearer, emphasize that it is the *change* in depressive symptoms between baseline and follow-up, which is compared between the two groups, e.g., “To compare the *changes in* depressive symptoms…”.
- The two sentences before the aims (“We designed a cluster RCT…”) and the two below the aims (“This study protocol is a cluster RCT…”) are both related to the methods and should be moved (and merged, as they repeat the same information) from the section “Background” to the section “Methods”, for example as a sub-heading “Design” at the beginning of the section.

Methods:

- Study setting: The last two sentences (“To obtain the sample…”) should be moved to the section “Recruitment”, as it is confusing here how centers were identified and matched.
- Study setting: For international readers, it would be informative with a short description of the healthcare system in Chile, i.e. are the primary care clinics public or private and free of charge or with user payment.
- Eligibility criteria: Are there any exclusion criteria related to e.g., comorbidity, alcohol or substance misuse, somatic illness?
- Interventions: The first sentence (“Eligible primary care clinics will…”) should be moved to the section “Recruitment”. It would also make more sense when reading, if the section “Recruitment” came before this section on “Interventions”, and if the heading was changed to “Recruitment and randomization”.
- Intervention arm: The intervention could be described in more details, as each element is not clearly described, but merely listed. For example, what is the role of the case manager (scheduled meetings with the patient and/or the primary care team? Inter-professional communication? Monitoring of follow-up assessments?), and how are the individual treatment plans developed (based on which instructions/guidelines or principles of care? Stepped care algorithm? Treatment modalities?).
- Outcome measures: Emphasize that the *change* in symptoms between baseline and follow-up is the measure: “Difference in depressive symptoms *change*”. Same for the secondary outcomes.
- All about blinding and randomization must be move to a separate section called “Blinding and randomization”. Together with the already given information, the authors need to address the fact that all the outcomes are self-report, which can lead expectation bias.   
- Recruitment: When is the recruitment expected to end (length of inclusion period)?
- Monitoring: Here the authors write “ These patients will be excluded from the study as well as patients who do not want to continue with the evaluation. Are the patients excluded from the treatment or the study as a whole. If the first change the wording, if he last, remove the phases about using intention-to treat under “data analysis”, as this is not the case.  

References: Please check the reference list carefully, as:

- Several of the references link to URLs that does not exist.
- Some institution names are incorrect.
- Links to web pages should list access date or publication date.
- Reports and documents should list name of institution/publisher and location.

Formatting: There are several phrasing and grammatical errors, which confuse the reading, such as:

- Phrasing: In the title, the term “on the *resolution* of adult depression” is unspecific. Replace with “on the *treatment* of”.
- Proofreading: Please carefully proofread the entire paper again, as there are several errors, such as a word missing in a sentence or an extra word at the end of a sentence, which should be deleted.
- Grammatical errors: Please carefully go through the text and check for grammatical errors, as there are quite a few of these, such as the word “The” missing, or plural “s” missing, or confusion between are/is.
- *Efficacy versus effect are used interchangeably, please use the correct wording in all places. *

**Conclusion**

 The manuscript needs major revision before it can be indexed.

Is the study design appropriate for the research question?

Yes

Is the rationale for, and objectives of, the study clearly described?

Yes

Are sufficient details of the methods provided to allow replication by others?

Partly

Are the datasets clearly presented in a useable and accessible format?

Not applicable

Reviewer Expertise:

psychiatry, mental health, recovery, interventions, implementation, collaborative care

We confirm that we have read this submission and believe that we have an appropriate level of expertise to confirm that it is of an acceptable scientific standard, however we have significant reservations, as outlined above.

# Response

**Published:** 2024-09-27

**Authors:** VERONICA VITRIOL

**Affiliations:** Universidad de Talca, Chile

**Dear Reviewers,**

Lene F Eplov and Marte Ustrup

 We would like to extend our sincere gratitude for your thoughtful and detailed feedback on our manuscript. Your comments and suggestions have been instrumental in improving both the structure and content of the paper. We have carefully considered each of your points and made the necessary revisions to enhance the clarity and overall quality of the manuscript.

 Please note that this manuscript was originally submitted in 2021, prior to the commencement of the project. Since then, the study has been completed, and we are now in the process of publishing the results in the *Annals of Family Medicine*.

 Below, we have provided specific responses to your comments:

**Primary Outcome in the Abstract**

**Comment:** The primary outcome is not described in the “Methods” section of the abstract.

**Response:** We have added a description of the primary outcome in the "Methods" section of the abstract. Both the primary and secondary outcomes are now clearly outlined in the Methods section of the main text for better clarity.

**Background**

**Comment:** The focus in the Background section on a specific subgroup of people with Major Depression (MD) is unnecessary, as the study focuses on the entire population with MD in primary care.

**Response:** Thank you for this suggestion. We have revised the introduction to focus on the general population with MD, aligning with the study's objective of evaluating the intervention for all individuals with MD in primary care, rather than emphasizing a specific subgroup.

**Methods**

**Comment:** The “Modifications” section is unclear and misplaced.

**Response:** We have removed the “Modifications” section and incorporated relevant details into other sections for improved clarity and coherence.

**Comment:** It is unclear if the sample size calculation takes the cluster design into account.

**Response:** In this version, we have elaborated on the sample size calculation, including assumptions such as equal-sized clusters and intra-class correlation (ICC).

**Comment:** It is unclear how data analysis accounts for clustering of individuals within clinics.

**Response:** The data analysis section now clearly explains how clustering is handled, including conducting an intra-cluster correlation prior to the regression analyses.

**Minor Issues**

**Comment:** The "Discussion" section reads more like a conclusion.

**Response:** We have rephrased the "Discussion" section to reflect that the trial is ongoing, using language such as "This trial will provide information on whether...".

**Comment:** Please include the registration number for the trial.

**Response:** The trial registration number and date have been added at the end of the abstract.

**Hypotheses and Aims**

**Comment:** Only list aims and hypotheses related to the primary outcome.

**Response:** The aims and hypotheses have been revised to focus on those related to the primary and secondary outcomes. Aims such as designing the intervention (aim 1) have been removed unless explicitly described.

**Comment:** Change "improve depressive symptoms" to "a reduction in depressive symptoms."

**Response:** This wording change has been implemented for greater specificity.

**Comment:** Clarify that the aim is to compare changes in depressive symptoms between baseline and follow-up.

**Response:** The aim now explicitly states that we compared changes in depressive symptoms between baseline and follow-up in the two groups.

**Comment:** Sentences regarding the study design should be moved from “Background” to “Methods.”

**Response:** We have moved and merged the relevant sentences under a new sub-heading titled "Design" in the Methods section for clarity and to avoid repetition.

**Study Setting**

**Comment:** Move the last two sentences regarding recruitment to the “Recruitment” section.

**Response:** These sentences have been moved to the "Recruitment" section for better organization.

**Comment:** Provide a brief description of the healthcare system in Chile.

**Response:** A brief description of Chile’s healthcare system, including the public/private structure and costs to users, has been added.

**Comment:** Are there any exclusion criteria related to comorbidity, substance misuse, or somatic illness?

**Response:** Exclusion criteria for this protocol were  bipolar disorder, suicide attempt, or severe drug dependence and consequently, they have been included in the updated version.

**Interventions**

**Comment:** The intervention could be described in more detail.

**Response:** The intervention arm has been elaborated, with a clearer description of the role of the case manager and the development of individual treatment plans.

**Outcome Measures**

**Comment:** Emphasize that the measure is the change in symptoms between baseline and follow-up.

**Response:** We have clarified that the measure is the change in depressive symptoms between baseline and follow-up, and similar wording has been applied to secondary outcomes.

**Comment:** All details regarding blinding and randomization should be in a separate section.

**Response:** A separate section titled "Blinding and Randomization" has been added, addressing the use of self-reported outcomes and the potential for expectation bias. Additionally, patients will be followed in a blinded manner by residents administering the follow-up surveys, which will be independent of self-reported information provided to the treatment team.

**Recruitment**

**Comment:** When is recruitment expected to end?

**Response:** The recruitment timeline, including the expected end date and the length of the inclusion period, has been specified.

**Monitoring**

**Comment:** Clarify whether patients are excluded from treatment or the study as a whole. **Response:** Patients may discontinue treatment or evaluations but will still be included in the analysis based on the intention-to-treat principle. The wording has been updated to reflect this distinction.

**References**

**Comment:** There are several issues with the reference list.

**Response:** The reference list has been reviewed and updated. All URLs have been checked for accuracy, institutional names have been corrected, and links to web pages now include access or publication dates. Reports and documents list the appropriate institution or publisher and location.

**Formatting**

**Comment:** There are phrasing and grammatical errors, and the terms "efficacy" and "effect" are used interchangeably.

**Response:** The manuscript has been thoroughly proofread to correct phrasing, grammatical errors, and consistency issues. The terms “efficacy” and “effect” have been replaced with “effectiveness,” where appropriate, to ensure proper usage.

 Once again, we are grateful for your time and valuable input, which have greatly improved the manuscript. We hope the revised version meets your expectations.

                                                                                  Sincerely,

                                                                          Dr. Veronica Vitriol

                                                                           Profesor of Psychiatry

   27/Sept/2024                                                      Universidad de Talca

# Reviewer report: Reviewer response for version 1

**DOI:** 10.5256/f1000research.79682.r188474

**Published:** 2023-07-31

**Authors:** Paula Dagnino

**Affiliations:** 1 Psychology, Universidad San Sebastian, Concepción, Bío Bío Region, Chile; 2 Millennium Institute for Research on Depression and Personality, Santiago, Chile

I emphasize the importance of this study. It considers a central pathology, such as depression, specifically patients who are difficult to treat. The fact that the study will include complementary variables to the symptomatology, such as emotional regulation, comorbidity, interpersonal relationships, etc., is in the most updated line of research in this field, in personalized or patient-centered treatment. The training of health professionals in Chile needs to be improved in this type of complexity and also in interdisciplinary, community, and network treatment. Knowing that this is "the" way of treating patients, the CMAD protocol will significantly contribute to primary care.

 The document is well-written and clear in each of the sections. I will be looking forward to the results.

Is the study design appropriate for the research question?

Yes

Is the rationale for, and objectives of, the study clearly described?

Yes

Are sufficient details of the methods provided to allow replication by others?

Yes

Are the datasets clearly presented in a useable and accessible format?

Yes

Reviewer Expertise:

Research in clinical psychology. Research in Depression and Personality. Research in patients treatment

I confirm that I have read this submission and believe that I have an appropriate level of expertise to confirm that it is of an acceptable scientific standard.
